# SGLT1 Inhibition Attenuates Apoptosis in Diabetic Cardiomyopathy via the JNK and p38 Pathway

**DOI:** 10.3389/fphar.2020.598353

**Published:** 2021-01-12

**Authors:** Na Lin, Hui Lin, Qi Yang, Wenqiang Lu, Zhenzhu Sun, Shimin Sun, Liping Meng, Jufang Chi, Hangyuan Guo

**Affiliations:** ^1^ Department of Cardiology, Shaoxing People's Hospital (Shaoxing Hospital, Zhejiang University School of Medicine), Shaoxing, China; ^2^ Department of Cardiology, The First Clinical Medical College, Wenzhou Medical University, Wenzhou, China

**Keywords:** SGLT1, apoptosis, diabetic cardiomyopathy, H9C2, DCM

## Abstract

**Background:** Recent studies have revealed that a novel selective sodium-glucose cotransporter 1 (SGLT1) inhibiton has shown beneficial effects in cardiovascular diseases. However, the question of whether SGLT1 inhibition influences diabetic cardiomyopathy (DCM) remains unanswered. In this study, we investigated the influence and underlying mechanism of SGLTI inhibition on DCM.

**Methods:** SGLT1 levels were measured in diabetic patients with similar conditions who visited our hospital from January to December 2019. Wistar male rats (n = 50) were divided into five groups: control, diabetes induced by streptozotocin infusion, and diabetes treated with 0.5, 1.0, or 1.5 mg/kg mizagliflozin via stomach gavage for 12 weeks. H9C2 cardiomyocytes were treated with mizagliflozin and then exposed to a high glucose concentration (30 mmol/L). TUNEL assays were performed, and bcl2, bax, p-p38, p-Erk, p-JNK and caspase-3 levels were measured. We used siRNA and an SGLT1 overexpression plasmid to detect the effects of SGLT1.

**Results:** SGLT1 levels were significantly elevated in DCM patients, and receiver operating characteristic (ROC) curve analysis identified SGLT1 as influencing DCM. The area under the curve (AUC) was 0.705 (*p* < 0.05), with 65.8% sensitivity, and 62.2% specificity. SGLT1 inhibition appeared to attenuate apoptosis in DCM via the JNK and p38 pathway.

**Conclusion:** SGLT1 can be used as a marker for the diagnosis of DCM, and SGLT1 inhibition can attenuate apoptosis, thereby suppressing DCM development via the JNK and p38 pathway.

## Introduction

Diabetic cardiomyopathy (DCM) is a myocardial disease specific to patients with diabetes. It is independent of other types of heart disease, including hypertension, coronary disease, and valvular heart disease (Bugger and Abel, 2014; Seferovic and Paulus, 2015). The causes of DCM remain unclear; however, oxidative stress, inflammation, autophagy, apoptosis, and endothelial dysfunction have been associated with DCM (Parim et al., 2018). Further studies are needed to explore the underlying mechanisms of DCM development.

Sodium-glucose cotransporters (SGLTs) belong to the solute carrier 5 (SLC5) gene family (Wood and Trayhurn, 2003; Sano et al., 2020). The SLC5A family in humans includes 12 genes, six of which are expressed in the heart. SGLT1 and SGLT2, encoded by the genes SLC5A1 and SLC5A2, respectively, have recently attracted much attention. SGLT1 mRNA has been detected in various tissues in humans, including the lung, heart, trachea, cervix, stomach, mesenteric adipose tissue, and pancreatic alpha cells. Recent experiments have also found that its expression is elevated in hypertrophic, ischemic, and diabetic cardiomyopathy in humans (Song et al., 2016). The use of KGA-2727, a selective SGLT1 inhibitor, has been demonstrated to be protective against myocardial infarction-induced ischemic cardiomyopathy (Sawa et al., 2020). Hirose et al. demonstrated that knock-out of SGLT1 alleviates pressure overload-induced cardiomyopathy, suggesting that SGLT1 inhibitors have an active effect on hypertrophic cardiomyopathy (Hirose, 2018). However, it is unclear whether SGLT1 has any influence on the development of DCM.

MAPKs, which include extracellular signal-regulated kinase 1/2 (ERK1/2), c-Jun N-terminal protein kinase (JNK), and p38 MAP kinase, regulate a range of physiological and pathological processes. JNK and p38 MAPK are major intermediates of apoptosis in DCM (Wang et al., 2016; Xu et al., 2016). We evaluated SGLT1 expression in patients with DCM, examined the effect of SGLT1 inhibition on the development of DCM, and investigated whether SGLT1 works through the JNK and p38 pathway.

## Materials and Methods

### Patients and Specimen Collection

This research was approved by the Ethics Committee of Shaoxing People’s Hospital, and informed written consent was obtained from all participants. We recruited 76 patients with diabetes, who were treated in our hospital from January to December 2019. Patients with obvious coronary stenosis, valvular, congenital, or pulmonary heart disease, hypertension, or liver or kidney dysfunction were excluded. All patients underwent echocardiography. All echocardiograms were performed by registered diagnostic cardiac sonographers and interpreted by an echocardiologist. DCM was diagnosed in patients who met all of the following criteria: 1) the presence of diabetes mellitus; 2) systolic or at least moderate diastolic dysfunction (systolic dysfunction was defined as an ejection fraction of <50%; moderate diastolic dysfunction was defined as advanced reduction in compliance or reversible or fixed restrictive filling, 0.75 < E/A<1.5, and E/e′≥10, severe diastolic dysfunction was defined as advanced reduction in compliance or reversible or fixed restrictive filling, E/A>1.5, E/e′≥10) (Redfield et al., 2003) after the diagnosis of diabetes mellitus; 3) no history of clinical heart failure; 4) no history of coronary disease; 5) no history of hypertension; 6) no history of significant valvular disease; and 7) no history of congenital heart disease (Dandamudi et al., 2014). Patients were divided into control and DCM groups using the diagnostic criteria for DCM. The two study groups were similar (Table 1), with no statistically significant differences in gender or age (*p* > 0.05). The patients were administered serum on an empty stomach. SGLT1 levels were detected using enzyme-linked immunosorbent assay (ELISA) kits. The diagnostic efficacy of serum SGLT1 in DCM was analyzed using receiver operating characteristic (ROC) curves.

**TABLE 1:** Patient characteristics.

	DCM	Control	*p* value
Age (years)	62.92 ± 12.66	64.76 ± 11.61	0.511
Male	24	19	
Female	14	19	0.247

### Animals

Sixty male Wistar rats, four-weeks-old, were purchased from Nanjing Biomedical Research Institute of Nanjing University, China, Animal procedures were performed according to the Guide for the Care and Use of Laboratory Animals from the National Institutes of Health, and approved by the Animal Care and Use Committee of Shaoxing People’s Hospital. All rats were kept in a 12-h light-dark cycle at 22°C. After acclimatization for 2 weeks, rats randomly divided into five groups (n = 10): control, diabetes, and diabetes treated with 0.5, 1.0, or 1.5 mg/kg mizagliflozin via stomach gavage. The control group received normal chow, while the other four groups were fed a high-fat (HF) diet, in which fat provided 60% of total calories (Research Diet D12492, HFD). After four weeks, we performed intraperitoneal insulin tolerance tests and intraperitoneal glucose tolerance tests to identify insulin-resistant rats. Rats fed a high-fat diet, and exhibiting insulin resistance, were administered a single intraperitoneal injection of streptozotocin (30 mg/kg, STZ dissolved in 0.1 mol/L sodium citrate buffer, pH 4.5). Control rats were injected with an equal volume of citrate buffer. Fasting blood glucose (FBG) levels were measured seven days after injection. Rats with FBG ≥ 11.1 mmol/L were considered to be a successful diabetic model (Ti et al., 2011; Wang et al., 2019). Then, the diabetic rats were respectively treated with mizagliflozin (SGLT1 inhibitor, gavage at 0.5, 1.0, 1.5 mg/kg), or vehicle (saline, gavage) once daily for 12 weeks. The cardiac function of the surviving rats was determined using echocardiography. The mice were then sacrificed, and their hearts and blood were obtained for use in subsequent experiments.

### Blood Analyses

After the rats were fasted overnight, blood samples were obtained from the jugular vein. Total cholesterol (TC), triglyceride (TG), FBG, and free fatty acid (FFA) levels were measured using the corresponding detection kits (Nanjing Jiancheng Bioengineering Institute, Nanjing, China). Fasting insulin (FINS) was measured using enzyme-linked immunosorbent assay (Nanjing Jiancheng Bioengineering Institute, Nanjing, China). The insulin sensitivity index (ISI) was calculated using the following formula: ISI = ln [([FBG] × [FINS])−1].

### Cell Culture and Treatments

The H9C2 cardiac myoblast cell line was purchased from Chinese Academy of Sciences Cell Bank (Shanghai, China) and cultured in DMEM (Sigma, Shanghai, China) with 10% FBS (Gibco, Shanghai, China) and 1% penicillin-streptomycin at 37°C in a 5% CO_2_ atmosphere. After cell confluence reached 80%, the cells were incubated with normal or high D-glucose (HG) concentrations (5.5 or 30 mM, respectively) combined with various concentrations of mizagliflozin (Sigma, Shanghai, China) for 24 h. To knock down SGLT1 in H9C2 cells, siRNA against SGLT1 and scrambled siRNA (synthesized by Shanghai MedChenExpress, Shanghai, China) were transfected using Lipofectamine 3000 Transfection Reagent (Invitrogen, Waltham, MA, United States) following the manufacturer’s instructions. The SGLT1 overexpression plasmid was transfected into H9C2 cells in the same manner.

### Histological Staining

Tissue specimens were fixed and dehydrated using a paraformaldehyde buffer solution, and embedded in paraffin. General tissue morphology was evaluated using hematoxylin and eosin (HE) staining, and myocardial fibrosis was examined using Masson staining (Beyotime, Nantong, China). Stained sections were visualized using a Nikon Eclipse Ti-U fluorescence microscope (Tokyo, Japan).

### Western Blotting

Rat heart tissue and H9C2 cells were lyzed using BCA protein. After adding 20 μg of sample to each well of the SDS-PAGE gel, gel electrophoresis was performed at 80 V for 30 min to stack the proteins, and then at 120 V for 1.0 h to resolve the protein bands. The protein bands were then transferred to a nitrocellulose membrane with a pore size of 0.22 µm, and run at 200 V for 1.5 h. After transfer, the membrane was washed with TBST three times at room temperature and blocked using skim milk powder for 1 h. Rabbit anti-mouse SGLT1, bcl2, bax, and caspase3, p38, p-p38, JNK, p-JNK, Erk, p-Erk (all obtained from Abcam Biotechnology, United States) antibodies were then added at a concentration of 1:1,000 and incubated at 4°C overnight. The target protein was detected by enhanced chemiluminescence after incubation with sheep anti-rabbit secondary antibody.

### RNA Analysis

Total RNA from tissue or cultured H9C2 cells was isolated using Trizol reagent (Invitrogen, Carlsbad, CA, United States) and reverse transcribed using PrimeScript Reverse Transcription Reagent Kits (Takara, Otsu, Japan). RT-PCR was performed using SYBR Premix Ex Taq Kits (Takara) on an ABI 7300 RT-PCR Detection System (Applied Biosystems, Foster City, CA, United States). The following primer sequences were used: SGLT1 forward, 5-GGA​CAG​TAG​CAC​CTT​GAG​C-3, and reverse, 5-CCA​ACA​GTC​CCA​CGA​TTA​G-3; Bax forward, 5-GGT​GGT​TGC​CCT​TTT​CTA​CTT​TGC-3, and reverse, 5-GCT​CCC​GGA​GGA​AGT​CCA​GTG-3; Bcl2 forward, 5-GGG​CTA​CGA​GTG-GGA​TAC​TGG​AG-3, and reverse, 5-GGG​CTA​CGA​GTG-GGA​TAC​TGG​AG-3; Caspase-3 forward, 5-ACT​GGA​AAG​CCG​AAA​CTC​TTC​ATC​A-3, and reverse, 5-GGA​AGT​CGG​CCT​CCA​CTG​GTA-TC-3; β-actin forward, 5-CCA​GAT​CAT​GTT​TGA​GAC​CT-3, and reverse, 5-TCT​CTT​GCT​CGA​AGT​CTA​GG-3′. Each group of samples was repeated three times.

### TUNEL Assays

After the cells in each group were treated in 96-well plates, TUNEL assays were carried out using Roche TUNEL staining kits (Roche, Basel, Switzerland), according to the manufacturer’s instructions, and images were captured using a fluorescence confocal microscope. The apoptotic rate was taken as the percentage of TUNEL-positive cells.

### Caspase3 Activity

Treated cells from each group were collected, lyzed, and centrifuged to remove the supernatant. Samples were incubated with reaction solution (Solarbio, Beijing, China) and assessed using a microplate reader.

### Statistical Analysis

Statistical analyses were performed using GraphPad Prism five software (GraphPad Software, San Diego, CA, United States). Data are presented as the mean ± SEM. Differences between two groups were analyzed using t-tests, and data among multiple groups were compared using one-way analysis of variance followed by Tukey’s post-hoc analysis. *p* < 0.05 was considered statistically significant.

## Results

### Diagnostic Value of SGLT1 for DCM

**FIGURE 1:**
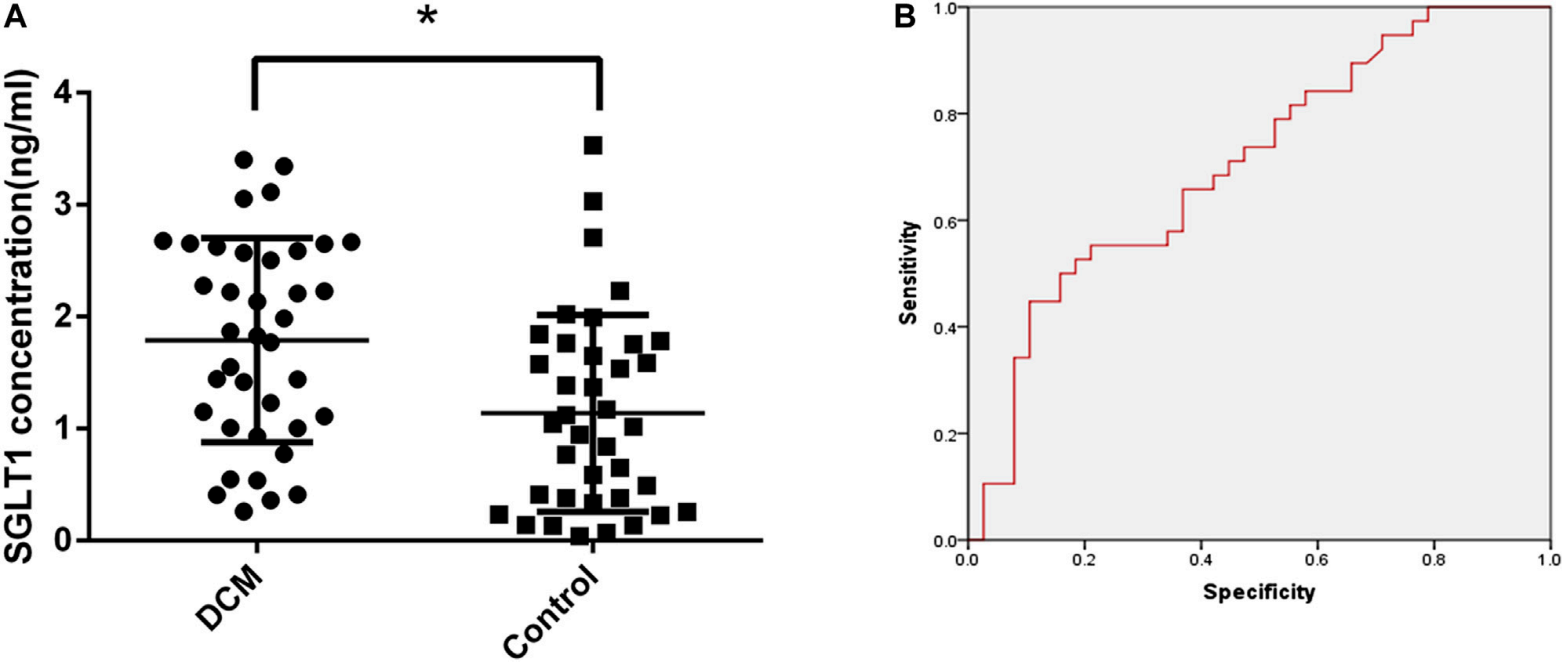
Diagnostic value of SGLT1 for DCM. **(A)** SGLT1 level in DCM and diabetic patients. *p < 0.05 compared with the control group. **(B)** ROC curve.

After collecting patient serum samples, serum SGLT1 expression was detected by ELISA. A statistically significant difference (*p* < 0.05) in SGLT1 was observed between the control and DCM groups (Figure 1A). The area under the curve (AUC) of the ROC curve was 0.705 with 65.8% sensitivity and 62.2% specificity, which was considered potentially indicative of DCM (Figure 1B). To further investigate the correlation between SGLT1 and DCM, we conducted a logistic regression analysis. Using the ROC curve, we identified 1.40 ng/mL as the threshold value associated with DCM outcomes. All patients were divided into high-SGLT1 (≥ 1.40 ng/ml) and low-SGLT1 (< 1.40 ng/ml) groups. SGLT1 was significantly associated with DCM (*p* = 0.013, odds ratio = 3.297, 95% confidence interval 1.288–8.440). These results suggest that SGLT1 has clinical utility for DCM diagnosis.

### Basic Characteristics of Rats with Type 2 Diabetes

**FIGURE 2:**
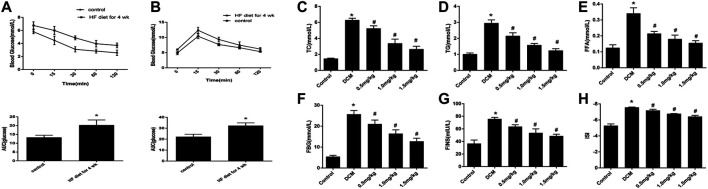
Changes in metabolic indices in rats (n = 10). **(A)** IPGTT and **(B)** IPITT were conducted in rats after four weeks of a HF diet. **(C)** TC, (**D**) TG, **(E)** FFA, **(F)** FBG, **(G)** FINS, and **(H)** ISI were measured using test kits. IPGTT, intraperitoneal glucose tolerance test; IPITT, intraperitoneal insulin tolerance test; TC, cholesterol; TG, triglyceride; FFA, free fatty acid; FBG, fasting blood glucose; FINS, fasting insulin; ISI, insulin sensitivity index. Values are mean ± SEM. *p < 0.05 compared with control group; ^#^ *p* < 0.05 compared with DCM group.

IPITT and IPGTT were conducted after feeding rats an HF diet for four weeks. The HF group showed insulin resistance, and the AUC increased compared with that of the control group (Figures 2A,B). At the end of the experiment, we found that the heart weight, TC, TG, FFA, FBG, FINS, and ISI in the HF group were significantly higher than those in the control group, and these changes were attenuated after treatment with mizagliflozin (s 2C-H).

### SGLT1 Inhibition Improved Heart Function in Diabetic Rats

**FIGURE 3:**
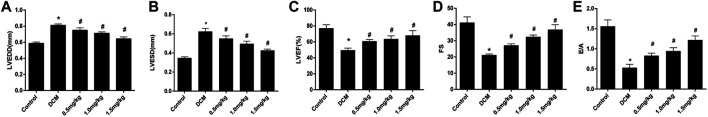
Mizagliflozin improved cardiac function in diabetic hearts (n = 10). LVEF, left ventricular ejection fraction; LVFS, left ventricular systolic function; LVEDD, left ventricular end-diastolic dimension; LVESD, left ventricular end-systolic dimension. Values are mean ± SD, *p* < 0.05. *p < 0.05 versus control; ^#^ *p* < 0.05 versus DCM.

As shown in Figure 3, the cardiac function of diabetic rats was impaired by left ventricular systolic and diastolic dysfunction compared with the control group. These pathologies manifested in the indicators of left ventricular ejection fraction (LVEF), left ventricular systolic function (LVFS), left ventricular end-diastolic dimension (LVEDD), left ventricular end-systolic dimension (LVESD), and E/A ratio. Following mizagliflozin supplementation, these changes were markedly improved in a concentration-dependent manner.

### SGLT1 Inhibition Relieved Myocardial Fibrosis and Apoptosis in the Hearts of Diabetic Rats

**FIGURE 4:**
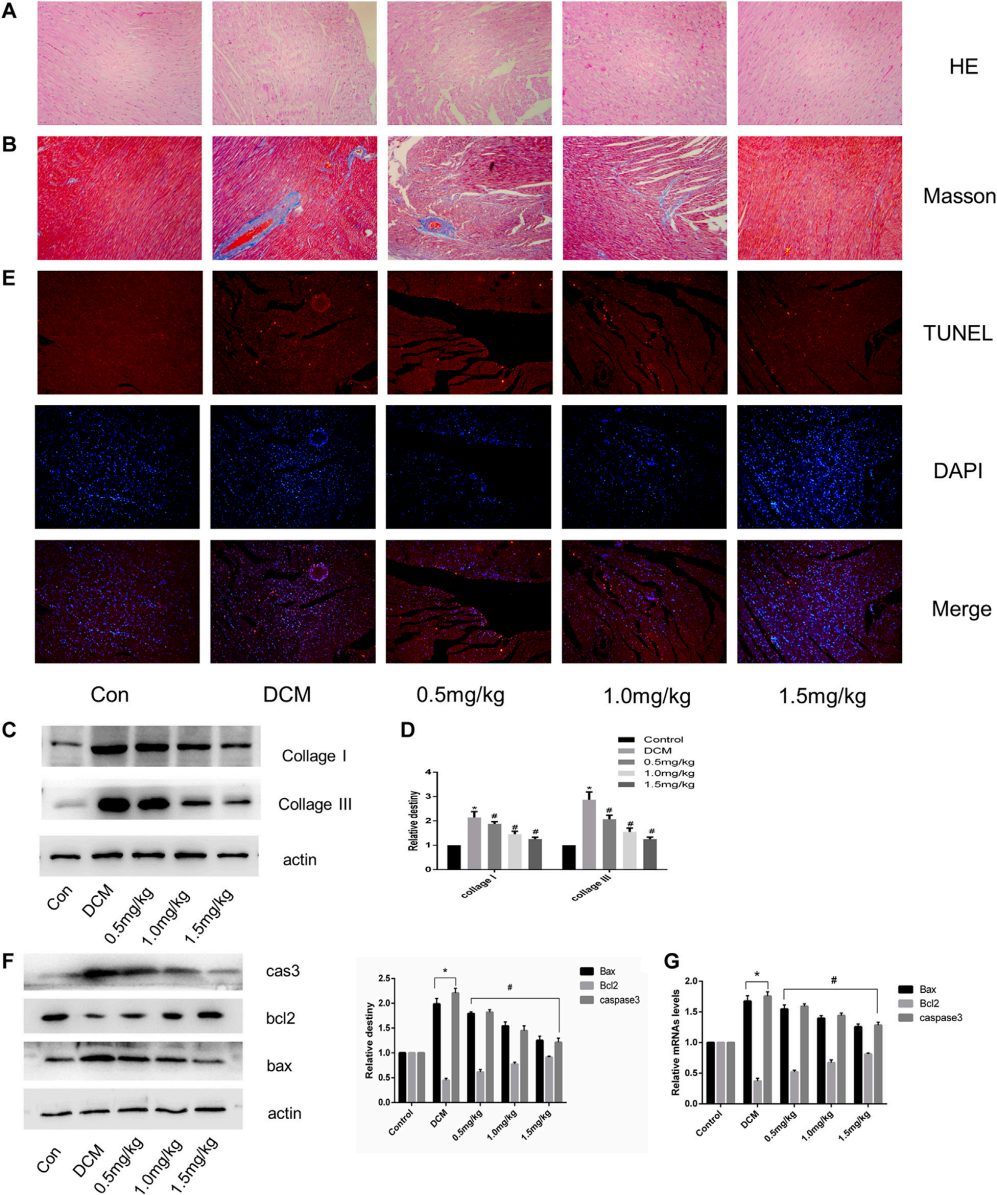
SGLT1 inhibition relieved myocardial fibrosis and apoptosis in diabetic rat hearts (n = 10). **(A–B)** HE and Masson’s Trichrome staining of myocardial tissue. **(C–D)** Relative protein levels of collagen I and collagen III. **(E)** Apoptotic rate of cardiomyocytes measured by TUNEL staining. **(F)** Bax, bcl2, and caspase-3 protein expression in rats. **(G)** RT-PCR analysis of bax, bcl2, and caspase-3 in tissues. *p < 0.05 control; ^#^ *p* < 0.05 vs. DCM. Experiments were performed in triplicate.

HE and Masson staining showed cardiac fibrosis with destroyed and irregular collagen network structure in the interstitial and perivascular areas in STZ-induced diabetic rats compared with normal rats (Figures 4A,B). The expression of fibrosis-related proteins, including collagen I and collagen III was significantly increased in the DCM group (Figures 4C,D). In contrast to the control group, diabetic rats showed a significant increase in TUNEL-positive cells (Figure 4E). In the DCM group, the expression of apoptosis-related genes such as cleaved caspase three and Bax decreased, accompanied by upregulation of the antiapoptotic gene Bcl-2 (Figures 4F,G). However, myocardial fibrosis and apoptosis were both notably alleviated in hearts from the SGLT1 inhibition group.

### SGLT1 Expression in H9C2 Cells After HG Stimulation and in Diabetic Rats

**FIGURE 5:**
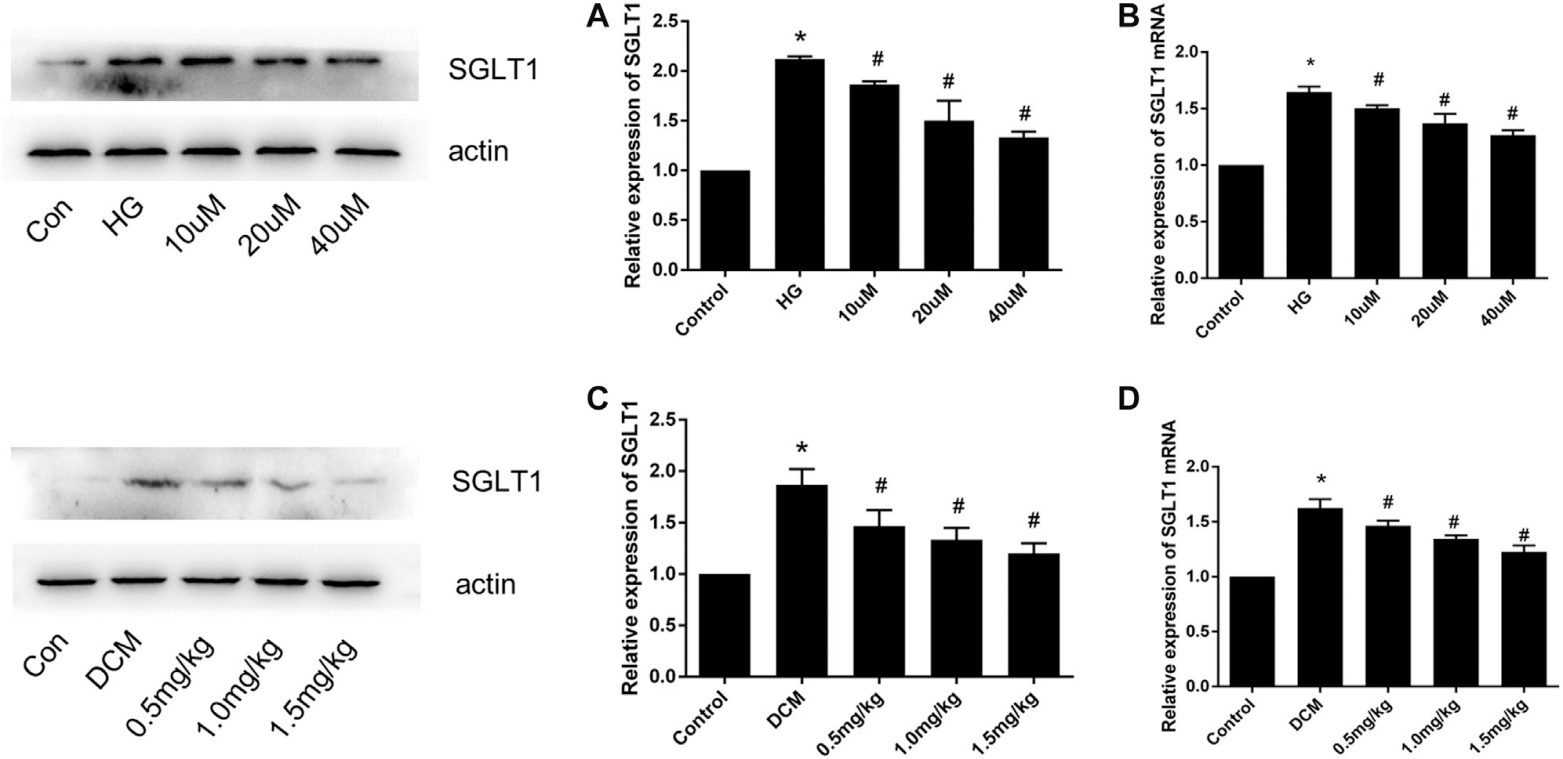
SGLT1 expression in H9C2 cells and diabetic rats (n = 10). **(A)** Western blot and **(B)** RT-PCR analyses of SGLT1 protein in H9C2 cells. **(C)** Western blot and **(D)** RT-PCR analyses of SGLT1 in tissues. *p < 0.05 compared with the control group; ^#^ *p* < 0.05 compared with the DCM group. Experiments were performed in triplicate.

To investigate the effect of SGLT1 on DCM, we assessed SGLT1 levels in H9C2 cells and heart tissues. As shown in Figures 5A,B, western blotting and PCR analyses revealed that SGLT1 expression was increased in the HG group compared with the control group of H9C2 cells, which was suppressed by mizagliflozin. Western blotting, PCR, and immunohistochemical assays revealed similar results in rat heart tissues (Figures 5C–E).

### SGLT1 Inhibition Relieved Apoptosis in H9C2 Cells

**FIGURE 6:**
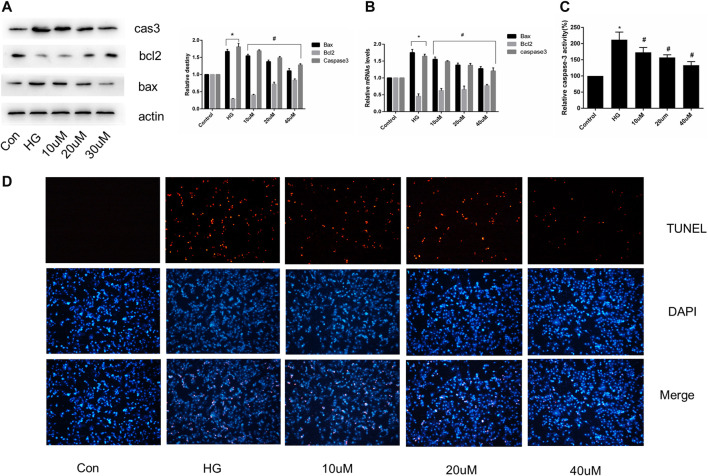
SGLT1 inhibition relieved apoptosis in H9C2 cells. H9C2 cells were treated with 10, 20, or 40 µM mizagliflozin for 24 h, followed by treatment with or without 30 mmol/L HG. **(A)** Relative protein levels of bax, bcl2, and caspase-3. **(B)** RT-PCR analysis of bax, bcl2, and caspase-3. **(C)** Caspase-3 activity in H9C2 cells. **(D)** Apoptotic rate of H9C2 cells measured by TUNEL staining. *p < 0.05 vs control; ^#^ *p* < 0.05 vs DCM. Experiments were performed in triplicate.

Cultured H9C2 cells were treated with or without mizagliflozin at various concentrations for 24 h, after which total RNA and protein were extracted. The expression of apoptosis-related mRNA and proteins, and the proportion of TUNEL-positive cells was significantly decreased in mizagliflozin-treated H9C2 cells compared with those in the HG group (Figures 6A–D).

### SGLT1 Up-Regulation Enhanced HG-Induced Apoptosis

**FIGURE 7:**
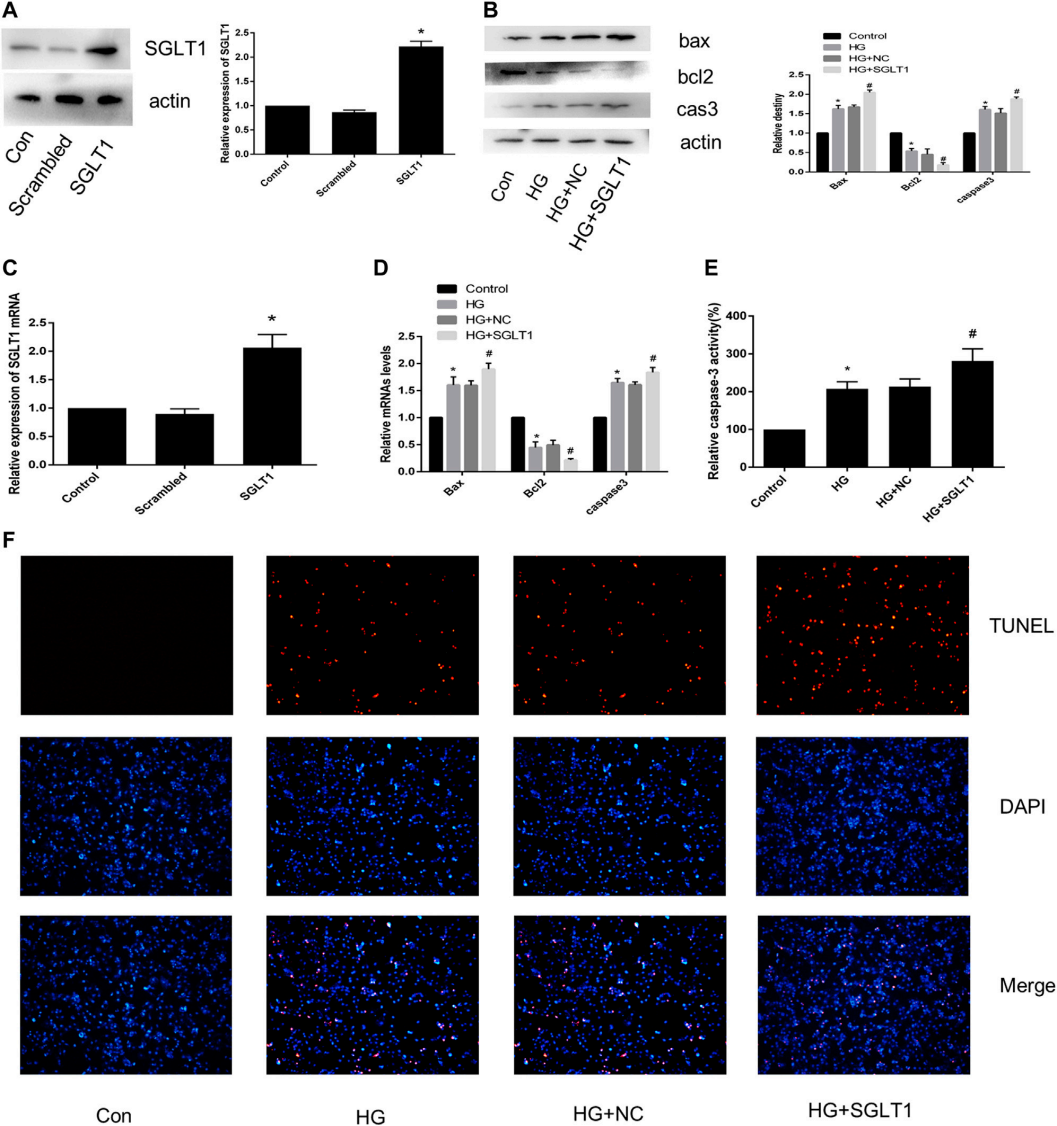
SGLT1 upregulation aggravated apoptosis induced by HG. **(A)** SGLT1 protein expression following successful transfection of SGLT1 into H9C2 cells. **(B)** RT-PCR analysis of SGLT1. **(C)** The expression of apoptosis-related specific markers (bax, bcl2, caspase-3). **(D)** Relative mRNA levels of bax, bcl2, and caspase-3. **(E)** Caspase-3 activity in H9C2 cells. **(F)** Apoptotic rate of H9C2 cells measured by TUNEL staining. *p < 0.05 compared with the control group; ^#^ *p* < 0.05 compared with HG group. Experiments were performed in triplicate.

Figures 7A,B show that the SGLT1 overexpression plasmid was successfully transfected into H9C2 cells. As shown in Figures 7C,D, SGLT1 upregulation resulted in an increase in apoptosis-related protein and caspase-3 activity compared to the HG group. RT-qPCR analysis of apoptosis-related specific markers, including bcl2, bax, and caspase-3, showed that up-regulation of Herp enhanced apoptosis (Figure 7E). TUNEL staining assays confirmed the increase in the rate of apoptosis in the SGLT1 group (Figure 7F).

### SGLT1 Down-Regulation Alleviated HG-Induced Apoptosis

**FIGURE 8:**
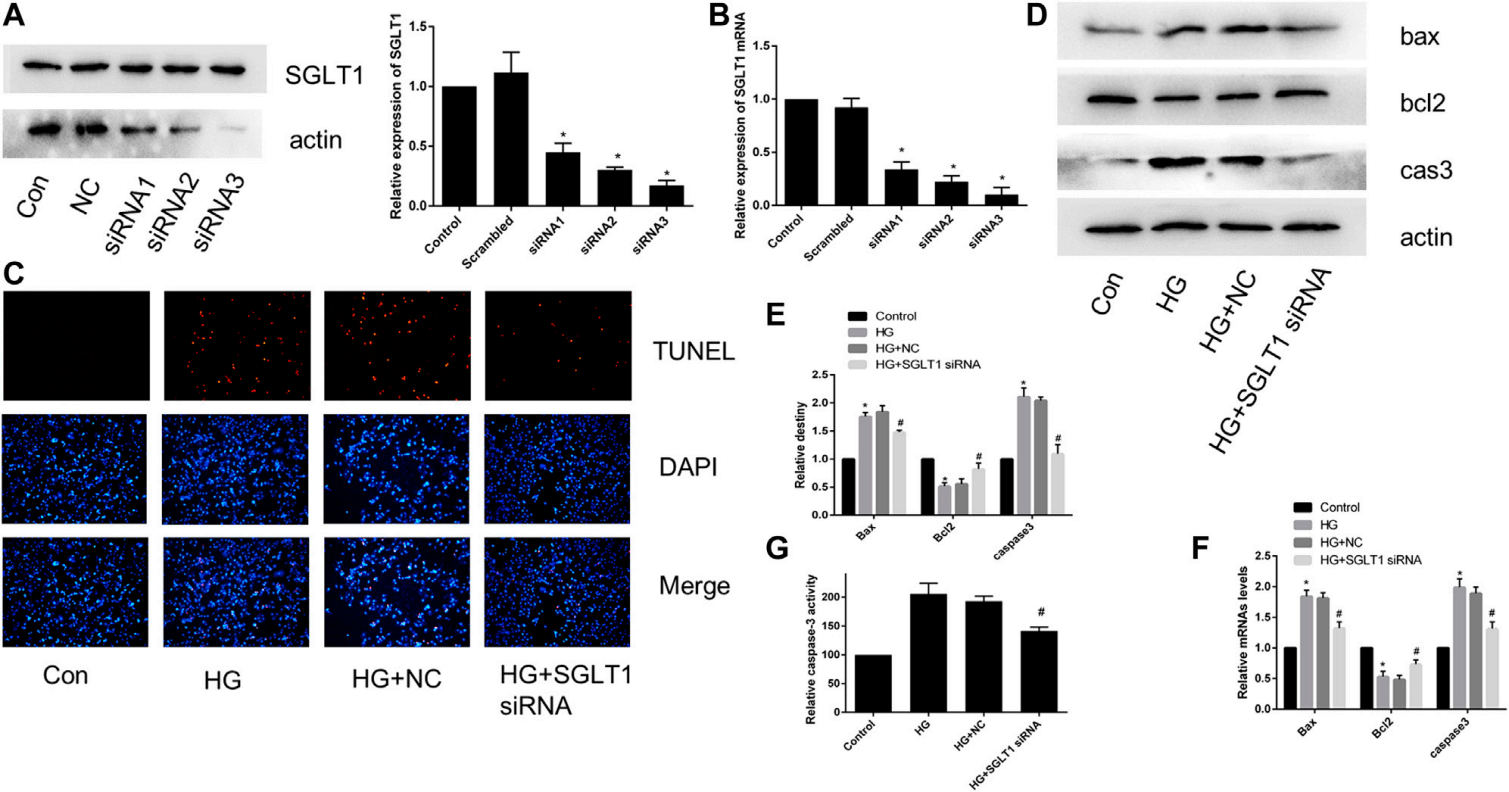
SGLT1 knockdown attenuated apoptosis induced by HG. **(A–B)** Specific siRNAs targeting SGLT1 were used to knockdown SGLT1, as confirmed by western blotting and RT-qPCR. **(C)** The apoptotic rate was determined by TUNEL assay. **(D–E)** The expression of bax, bcl2, and caspase-3 in H9C2 cells were detected by RT-PCR and western blot analyses. **(F)** Caspase-3 activity in H9C2 cells. *p < 0.05 compared with the control group; ^#^ *p* < 0.05 compared with HG group. Experiments were performed in triplicate.

To further confirm the effect of SGLT1 on H9C2 cells, we transfected SGLT1 siRNA into H9C2 cells and examined SGLT1 knockdowns using RT-qPCR and western blotting (Figures 8A,B). TUNEL staining showed that apoptosis increased with HG treatment (Figure 8C). HG increased the protein and mRNA expression of the apoptosis-related markers caspase-3 and Bax, and levels of the anti-apoptotic gene bcl2 were decreased (Figure 8D–F). However, SGLT1 siRNA markedly inhibited HG-induced apoptosis (Figures 8D–F).

### SGLT1 Inhibition Alleviated Apoptosis in DCM via the JNK and p38 Pathway

**FIGURE 9:**
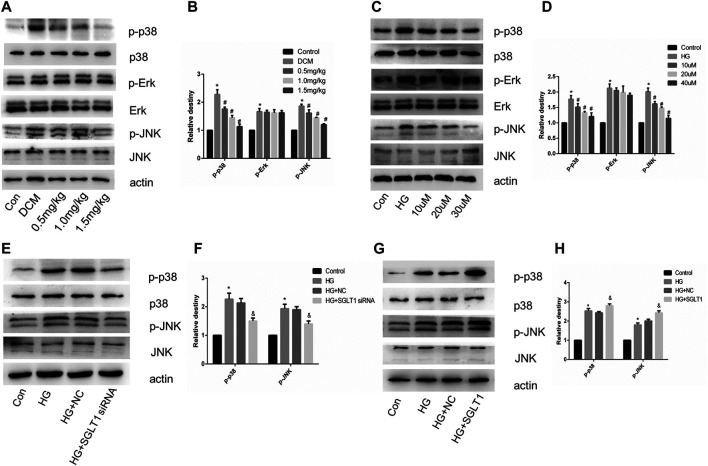
SGLT1 influenced apoptosis in DCM via the JNK and p38 pathway. **(A–B)** Expression of p-p38, p-Erk and p-JNK in rats (n = 10). *p < 0.05 compared with the control group; ^#^ *p* < 0.05 compared with the DCM group. Experiments were performed in triplicate. **(C–H)** Western blot of p-p38, p-Erk and p-JNK in H9C2 cells. *p < 0.05 compared with the control group; ^#^ *p* < 0.05 compared with HG group. Experiments were performed in triplicate.

We investigated the involvement of MAPK pathway in the regulation of apoptosis of cardiomyocytes via SGLT1 inhibition. *In vivo*, the ratio of p-JNK and p-p38 MAPK to their respective total proteins was significantly decreased in the SGLT1 inhibitor-treated group compared with the DCM group (Figures 9A,B). However, mizagliflozin had no significant effect on the phosphorylation of Erk1/2 *in vivo* and vitro (Figures 9A–D). In H9C2 cells, SGLT1 knockdown and SGLT1 inhibitor treated groups showed decreased expression of p-p38 and p-JNK (Figures 9C–F). Phosphorylation of JNK and p38 MAPK was observed to increase as a result of SGLT1 upregulation (Figures 9G,H).

## Discussion

Recently, diabetes mellitus has become an epidemic. Cardiovascular complications are the major cause of mortality and morbidity in diabetic patients (Murtaza et al., 2019). DCM is prevented and treated using two primary methods (Paolillo et al., 2019). One method involves suppressing the occurrence and development of DCM by lowering the blood glucose concentration and insulin resistance, using hypoglycemic drugs such as sulfonylurea, α-carbosidase inhibitors, biguanides, and insulin (Deedwania and Acharya, 2019). The other method involves using cardioprotective drugs, such as β-blockers and ACEI, to inhibit cardiac remodeling and improve heart function, thereby delaying the development of DCM (Bell and Goncalves, 2019). However, there are currently no drugs with both hypoglycemic and cardioprotective effects. In our previous research, we found that the serum SGLT1 levels of diabetic patients were significantly higher than those of non-diabetic patients, which prompted us to examine the role of SGLT1 in DCM. Therefore, our study aimed to explore the value of SGLT1 for the diagnosis of DCM, and the cardioprotective effect of SGLT1 inhibition on DCM development via JNK and P38 pathway.

SGLTs belong to the SLC5 gene family, which plays an important role in active glucose transport. SGLT1 and SGLT2 are the most studied members of this family (Wood and Trayhurn, 2003). Previously, SGLT1 was only found to be expressed in the brush border of the small intestine and the proximal tubules of the kidney, but not in other organs of the human body. Recently, however, Kashiwagi et al. reported that SGLT1 was highly expressed in cardiomyocytes, as assessed by immunostaining and immunoblotting (Kashiwagi et al., 2015). In our study, the results indicated that SGLT1 was overexpressed in H9C2 cells in rats as expected (Figures 5C–D). And mizagliflozin-treated rats showed lower blood glucose than DCM group rats, indicating that SGLT1 inhibition had a hypoglycemic effect (Figures 2F–H). Additionally, we found that SGLT1 has clinical utility for DCM diagnosis (Figures 1A,B).

With the development of DCM in diabetic patients, changes occur in the structure and function of the heart muscle, eventually leading to heart failure (The, 2018). Early changes in hearts with DCM include diminished cardiac diastolic function, and 40–75% of patients with type 1 or type 2 diabetes can exhibit abnormal diastolic function (Boyer et al., 2004; Shivalkar et al., 2006). We found that mizagliflozin improved cardiac function, with beneficial effects on LVEF, LVFS, LVESD, LVEDD, and the E/A ratio (Figures 3A–E). At the micro level, mizagliflozin alleviated the irregularity of myocardial fibers, organized the collagen network structure, and reduced collagen deposition (Figures 4A–D).

Numerous studies have indicated that cardiac cell apoptosis is the major event in DCM development (Li et al., 2007). Cardiomyocyte apoptosis in diabetic patients increased by 85-fold compared with non-diabetic patients, suggesting that cardiomyocytes are sensitive to apoptosis caused by diabetes (Ouyang et al., 2014). TUNEL staining revealed increased apoptosis in STZ-induced diabetic rats at 3 and 14 days. Therefore, inhibiting cardiomyocyte apoptosis is important for hindering DCM progression. Interestingly, we found that SGLT1 inhibition significantly decreased the level of TUNEL-positive cells and caspase-3, and the ratio of bax/bcl2 *in vivo* and vitro (Figures 4E–G, 6A–D). To further demonstrate the role of SGLT1 in DCM, overexpression plasmids were used to overexpress, and siRNA to downregulate, SGLT1. We found that SGLT1 overexpression in HG-stimulated H9C2 cells aggravated apoptosis, as evinced by an increase in apoptosis-related protein and mRNA levels and TUNEL-positive cells, whereas SGLT1 knockdown significantly inhibited HG-stimulated apoptosis in H9C2 cells (Figures 7A–F, 8A–G). These results confirm that SGLT1 inhibition ameliorates the impairment of cardiac function in DCM by improving apoptosis.

Among the mechanisms known to be associated with diabetes, the JNK and p38 pathway has been the most extensively evaluated (Kassan et al., 2014; Zuo et al., 2019). Hyperglycemia-induced ROS can activate MAPK to stimulate apoptosis in cardiomyocytes. In this study, the DCM group had increased phosphorylation of p38 and JNK, while SGLT1 inhibition attenuated p38 and JNK kinase activities *in vivo* (Figures 9A,B). The effects of high glucose on p38 and JNK kinase activities *in vitro* were also alleviated in the SGLT1 inhibition-treated group (Figures 9C,D). Overexpression and downregulation of SGLT1 were further used to demonstrate the connection at a genetic level between the JNK and p38 pathway and SGLT1 inhibition in DCM (Figures 9E–H).

Taken together, the data from this study suggest that SGLT1 has clinical utility for DCM diagnosis, and SGLT1 inhibition could attenuate apoptosis, to suppress DCM development via the JNK and p38 pathway, providing a promising treatment for DCM. Since age is a factor that plays an important role in the disease progression of DCM, the primary drawback of the current study is that this experiment was not modeled on aged rats corresponding to the elderly patients age. Another limitation is the lack of the group with only high-fat diet for comparison.

## Conclusion

The data suggest that SGLT1 has clinical utility for DCM diagnosis, and SGLT1 inhibition can attenuate apoptosis to suppress the development of DCM via the JNK and p38 pathway, potentially providing a promising treatment for DCM.

## Data Availability Statement

The original contributions presented in the study are included in the article/Supplementary Material, further inquiries can be directed to the corresponding authors.

## Ethics Statement

The studies involving human participants were reviewed and approved by Shaoxing Ethics Committee. The patients/participants provided their written informed consent to participate in this study. The animal study was reviewed and approved by Animal Care and Use Committee of the Shaoxing Hospital.

## Author Contributions

All authors listed have made a substantial, direct, and intellectual contribution to the work and approved it for publication.

## Funding

This research was supported by grants from the Social Development Project of Public Welfare Technology Application in Zhejiang Province (No.LGF19H020006) and the National Natural Science Foundation of China (No.81873120).

## Conflict of Interest

The authors declare that the research was conducted in the absence of any commercial or financial relationships that could be construed as a potential conflict of interest.
